# Exploring the development of asthma in children with parental and in utero exposure to smoking

**DOI:** 10.1186/1617-9625-12-S1-A10

**Published:** 2014-06-06

**Authors:** Eleni Xanthopoulou, Antonis A Kousoulis

**Affiliations:** 1 Society of Junior Doctors, Athens, 15123, Greece; 2 Medical School, National and Kapodistrian University of Athens, Athens, 115 27, Greece; 3 Centre for the History of Science, Technology and Medicine, Imperial College London, London, SW7 2AZ, UK

## Background

Second-hand smoking is a well-established asthma trigger and a risk factor for new asthma cases in infants. Aim of this review is to describe the effects of passive smoking exposure on children with asthma, as well as the impact of maternal smoking during pregnancy on the development of asthma in children.

## Materials and methods

We performed an electronic search in PubMed and Google Scholar and ginasthma.org using combinations of the main keywords: asthma, tobacco, smoking, and children. Our search was focused on the recent literature, between 2008 and 2013.

## Results

A significant number of studies have explored the topic in question in the recent years. There is increasing evidence that passive exposure to parental smoking is associated with asthma symptoms among children, as well as more severe disease among children with already diagnosed asthma. Children with asthma exposed to smoking at home tend to have more severe and frequent respiratory symptoms, more asthma-related doctor visits and a detrimental response to asthma therapy. Exposure to tobacco products diminishes asthma control, causing poorer response to inhaled corticosteroids, oral steroids and leukotriene receptor antagonists. Moreover, maternal smoking during pregnancy increases the risk of developing asthma. Newborns of mothers who have been smokers have altered cellular immune function and, as a result, decreased innate production of antigen-presenting cell cytokines and diminished response to TLR ligands. In utero-exposure to smoke causes additionally a predisposition to Th2-associated respiratory diseases and an increased risk for IgE-mediated sensitization.

## Conclusions

Second-hand smoking causes both a significant worsening and an increased number of asthma exacerbations in children with established disease. Moreover, it constitutes a risk factor for developing asthma in children with a predisposition to the disease. Finally, it seems to rank among the predisposing factors for asthma and other atopic diseases on offspring exposed to tobacco during fetal period.

